# Potential Role of High-Dose Steroids in the Treatment of COVID-19-Associated Nephropathy

**DOI:** 10.7759/cureus.35372

**Published:** 2023-02-23

**Authors:** Karishma Kadariya, Demilade Soji-Ayoade, Sangeetha Isaac, Subir Paul

**Affiliations:** 1 Internal Medicine, North Alabama Medical Center, Florence, USA; 2 Nephrology, North Alabama Medical Center, Florence, USA

**Keywords:** core renal biopsy, systemic steroids, collapsing glomerulonephritis, : acute kidney injury, covid 19

## Abstract

With the increasing number of COVID-19-associated nephropathy (COVAN), biopsy-proven cases of collapsing variety of focal segmental glomerulosclerosis (FSGS) are emerging. Though the recommendations on treatment for COVID-19-associated respiratory symptoms are evolving, there is still no definitive treatment for the collapsing FSGS secondary to COVAN. We report a case of a 47-year-old male admitted with acute kidney injury from COVID-19 infection and found to have collapsing FSGS on renal biopsy. Almost all the patients who were found to have similar conditions were treated with a relatively smaller dose of steroids and ultimately required dialysis. Our patient showed improvement with the trial of higher doses of steroids and never required dialysis. Hence, our case report emphasizes the need for a randomized controlled trial (RCT) with regard to the use of high-dose steroids in COVAN.

## Introduction

COVID-19 is a pandemic that started predominantly with respiratory manifestations. However, kidney involvement is now relatively frequent. Initial studies that were done in Wuhan, China, showed that up to 43.9% of patients presented with proteinuria and 26.7% with hematuria [1]. The prevalence of elevated serum creatinine, elevated blood urea nitrogen, and estimated glomerular filtration under 60 ml/min/1.73m2 were 14.4, 13.1, and 13.1%, respectively [1]. The studies also showed that the prevalence of kidney disease on admission and the development of acute kidney injury (AKI) during hospitalization in patients with COVID-19 is high and is associated with in-hospital mortality [1]. We all are aware of the association of collapsing variety of focal segmental glomerulonephropathy (FSGS) with diverse causes that include viral infections, mainly HIV infection [2]. There are emerging cases of collapsing FSGS with a temporal association with COVID-19 infection. In our case, we report one African-American patient who developed AKI in association with COVID-19 infection, who was found to have collapsing FSGS on renal biopsy, and the renal function improved with the treatment of long-term high-dose steroid. 

## Case presentation

A 47-year-old African-American male with a past medical history significant for chronic kidney disease stage II, renal cell carcinoma treated with cryoablation two years ago, type II diabetes mellitus, and essential hypertension presented to the ED with a 3-4 days history of nausea, non-bloody, non-bilious vomiting, anorexia, and non-bloody diarrhea. He also complained of a nonproductive cough and subjective feeling of fever and chills for a similar duration. Vitals were unremarkable, with a temperature of 98.5 Fahrenheit, pulse 98/minute(min), respiratory rate of 16/min, BP of 130/81 mm of Hg, and saturating 94% on room air. He was non-oliguric. Physical examination was unremarkable. Initial lab findings on admission are listed below in Table 1. 

**Table 1:** Lab values on admission. CBC: Complete blood count.

Labs	Reports
CBC	Unremarkable except relative lymphopenia
Blood urea nitrogen (BUN)	46 mg/dl
Creatinine	3.4 mg/dl
Glomerular filtration rate (GFR)	34 ml/min
Chest X-ray	Unremarkable
Spot urine analysis	Proteinuria >500 mg/dl
RT PCR for COVID-19	Positive

He was admitted with a diagnosis of COVID-19 and AKI, which was initially suspected to be pre-renal and managed accordingly with IV 0.9% normal saline. Initially, serum creatinine downtrended to 2.6 on the second day of admission. However, despite the correction of volume depletion and resolution of symptoms, serum creatinine started to rise. Urine analysis was subsequently repeated, which showed persistent proteinuria of >500 mg and no hematuria. Spot urine analysis, as well as bilateral renal ultrasound, was done that showed the following findings (Table 2).

**Table 2:** Results of spot urine analysis for protein and creatinine and bilateral renal ultrasound.

Spot urine analysis	Values
Spot urine protein	1900 mg
Spot urine protein creatinine ratio	27.9
Bilateral renal ultrasound	Normal-sized kidneys with bilateral increase echogenicity without hydronephrosis, stone or mass.

 A 24-hour urine study was done, the findings of which are presented in Table 3.

**Table 3:** Findings on 24-hour urine study .

24-hours urine	Values
Total volume	2 liters
Creatinine	12.48
Protein	12.80

**Figure 1:**
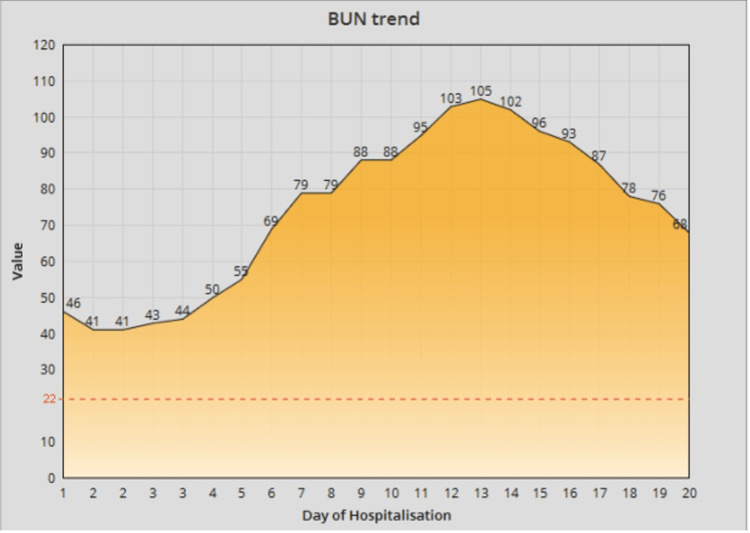
Serum blood urea nitrogen (BUN) trend.

**Figure 2:**
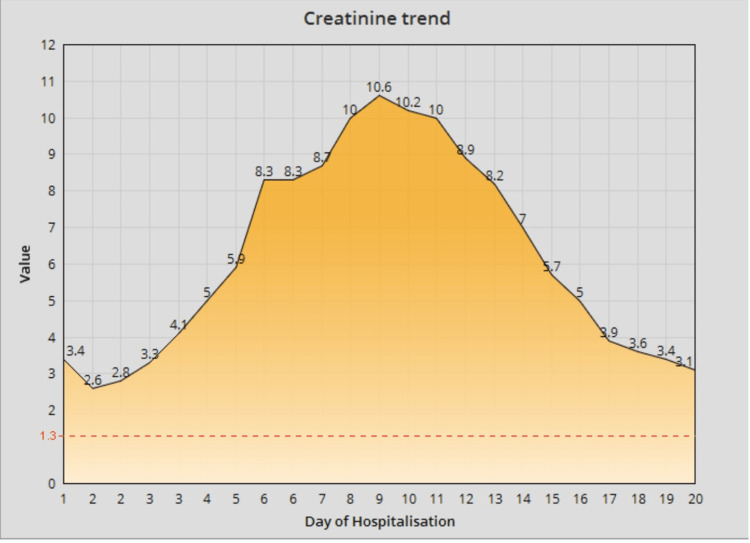
Serum creatinine trend.

Anti-nuclear antibody and anti-neutrophil cytoplasmic antibody vasculitis panel were negative. HIV screening was negative, and the viral hepatitis panel was non-reactive. Serum complement levels were normal. There was a progressive rise in serum BUN and creatinine, as presented in Figures 1-2.

**Figure 3:**
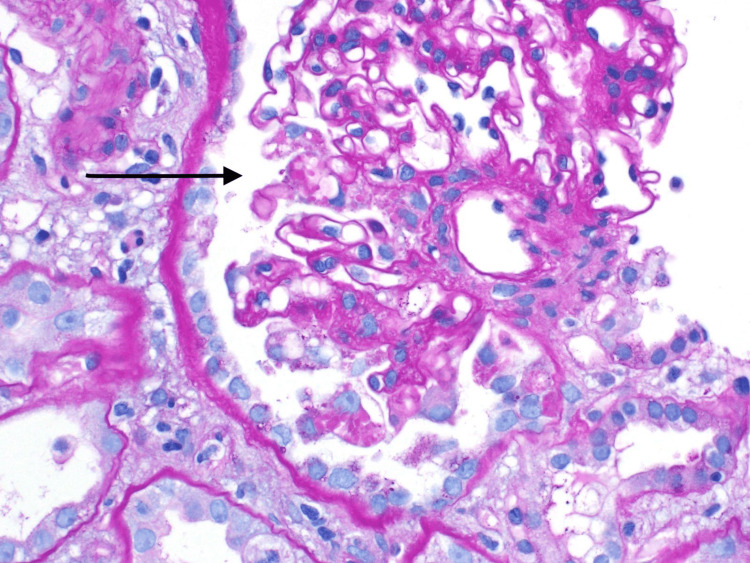
Arrow showing glomerular sclerosis.

**Figure 4:**
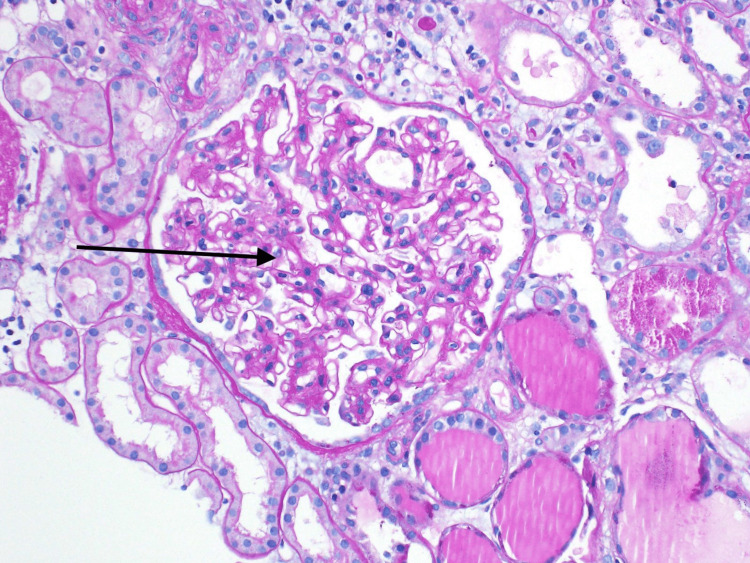
Arrow showing glomerulomegaly.

We then decided to perform the CT-guided renal biopsy on the seventh day of admission. Light microscopy showed up to fifteen glomeruli, out of which nine were globally sclerotic (Figure 3). The remaining glomeruli were found to be enlarged (Figure 4) and showed focal segmental mild mesangial matrix expansion with rare mild mesangial hypercellularity. Focal glomeruli showed the early segmental collapse of the capillary loops associated with overlying epithelial cell hypertrophy and prominent protein reabsorption droplets. There was no endocapillary hypercellularity, fibrinoid necrosis, or crescent formation. The glomerular basement membranes were without holes, spikes, or double contours on silver stain. Diffuse interstitial edema was associated with a moderate and predominantly mononuclear interstitial inflammatory infiltrate admixed with few eosinophils. Toluidine blue-stained sections showed two glomeruli with no global sclerosis. One glomerulus showed segmental collapse.

Immunofluorescence for IgG, IgM, IgA, C3, C1q, kappa and lambda light chains was performed on paraffin-embedded tissue after pronate digestion. All stains were negative within the glomeruli, though focal glomeruli showed prominent podocyte protein reabsorption droplets highlighted by most immunoreactants. There was no significant extra glomerular staining. Kappa and lambda were stained equally throughout the tubulointerstitium.

**Figure 5:**
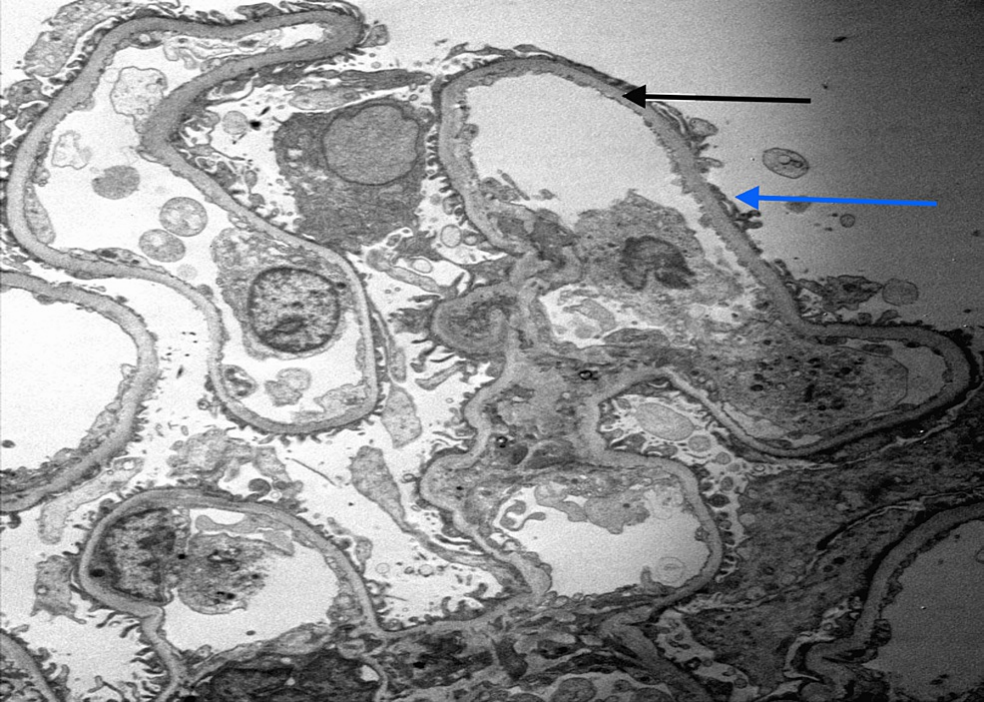
The black arrow showing the glomerular basement membrane thickening, and the blue arrow showing the effacement of the podocyte foot process.

Electron microscopy revealed uniform and mildly thickened basement membranes (Figure 5). There was a segmental area of capillary loop collapse. Glomerular capillary loops were otherwise patent. No immune-type electron-dense deposits were present along the glomerular basement membranes or within the mesangium. There was moderate segmental epithelial foot process effacement (Figure 5), and the tubular basement membranes were without deposits.

Apolipoprotein L1 genetic testing was considered; however, it could not be performed due to resource limitations. Serum creatinine continued to rise and peaked at 10.6 on day 9 of admission, following which 60 milligrams of IV methyl-prednisone every eight hours was started. Prior to this, the patient was steroid naïve. Serum creatinine started to a downtrend from the subsequent day and reached 5.7 mg/dl on day 5 of the initiation of steroid. Intravenous methyl-prednisone was transitioned to oral prednisone at 60 milligrams daily. Throughout the hospitalization, urine output remained adequate, and dialysis was never required. At discharge, creatinine was down to 3.1 mg/dl. The patient was followed up in the renal clinic. His renal function and proteinuria continued to improve. We added ramipril 5 milligrams daily. Blood pressure remained well controlled. Due to uncontrolled hyperglycemia and weight gain, we started to taper the prednisone. On the four-month follow-up, his estimated GFR was 51 ml/min, serum creatinine went down to 1.7 mg/dl, and urine protein creatinine ratio was 3.59. He was still on prednisone at that time.

## Discussion

COVID-19 virus initially emerged as a respiratory illness; however, soon, it was found to involve kidneys as well. As the cases of AKIs started rising, collapsing variety of FSGS associated with COVID-19 was also being recognized in postmortem reports from Wuhan, China. In April 2020, the first living case was recorded [3]. Now COVID-19-associated FSGS is emerging as a distinct global nephropathy. Despite the emerging cases of COVID-19-associated collapsing FSGS following ours, the definitive treatment remains uncertain, so we decided to report our case in which we were able to improve renal function without the need for hemodialysis. When we had our case of COVID-19 associated FSGS, there were only 4-5 cases reported prior to that. All of these cases ended up on hemodialysis ultimately. Since angiotensin-converting enzyme inhibitors (ACEIs) have shown remarkable results in HIV-induced collapsing FSGS [4], initially, we decided to give a trial of ACEIs in our patient as well. However, it had to be discontinued due to the progressive rise of serum creatinine as a side effect. Although not studied in a large randomized controlled trial (RCT), there is a study done by Smith MC et al. that has shown the beneficial role of steroid therapy in HIV-associated nephropathy characterized by collapsing variety of FSGS [5], so we decided to give a trial of high-dose steroids in our case as well. Two similar cases of COVID-19-associated collapsing FSGS before our case were treated with a relatively low dose of steroid; however, there was no improvement, and ultimately both patients needed hemodialysis [6]. Our patient started to show a gradual improvement in proteinuria from the second day of initiating IV steroids, and it continued to improve. He never required hemodialysis throughout the hospitalization. He was never treated with either remdesivir or tocilizumab since his respiratory status always remained stable, and he never required supplemental oxygen. So besides steroids, there has been no other medicine that can be attributed to his improvement. He was maintained on a high dose of steroids on discharge. On subsequent follow-ups, the dose was eventually tapered over six months. His serum creatinine and eGFR subsequently reached the baseline. There were minor side effects of long-term steroids, which were managed medically. Therefore, with this encouraging result in our patient, we hypothesize that a high dose of steroids would be beneficial in the treatment of COVAN, and randomized controlled studies are warranted. This can play an important role in preventing hemodialysis and its associated morbidities.

## Conclusions

Like other viruses, particularly HIV, COVID-19 infection can also lead to collapsing FSGS. At this moment, even though we do not have a definitive treatment, we propose that high-dose steroids can be used with a resultant favorable outcome. With the use of high doses of steroids, such nephropathy can improve, and the patients may be able to avoid dialysis-associated morbidities. Also, with our case and the remarkable outcome that was observed within a few months of the follow-up of the patient, we emphasize the need for RCTs in regard to this.
